# Giant subdural empyema following ventriculo-peritoneal shunt in a child

**DOI:** 10.11604/pamj.2017.26.120.11866

**Published:** 2017-03-02

**Authors:** Prastiya Indra Gunawan, Wihasto Suryaningtyas

**Affiliations:** 1 Department Of Child Health, Airlangga University, College of Medicine, Dr Soetomo Hospital, Surabaya, Indonesia; 2 Pediatric Neurosurgeon, Department Of Neurosurgery, Airlangga University, College of Medicine, Dr Soetomo Hospital, Surabaya, Indonesia

**Keywords:** Giant subdural empyema, child, ventriculo-peritoneal shunt

## Image in medicine

Subdural empyema is an intracranial focal collection of purulent material located between the dura mater and the arachnoid mater. It developed from varied sources, but the paranasal sinuses, the ears, and the mastoids processes were predominantly affected. Giant subdural empyema secondary to cerebrospinal fluid shunt placement has been extremely unusual. A 9-years-old girl presented with prolonged low-grade fever, vomiting, local wound infection in frontal area and general weakness. She was previously hydrocephalic with large head size since birth. A ventriculo-peritoneal shunt was already inserted for 7 years. Neurological examination showed the patient fully conscious and left hemiparesis. Routine hematological investigation revealed leukocytosis and high C-reactive protein (CRP). A contrast-enhanced CT scan showed an hypodense fluid collection with peripheral ring enhancement on the right of the midline supporting a giant subdural empyema. Emergency surgery was performed to drainage the pus. Pus culture resulted no growth of bacteria. Ceftriaxone and metronidazole were administered for 6 weeks. Follow-up CT Scan showed reduction of mass effect. The patient was improved leaving neurological sequalae.

**Figure 1:**
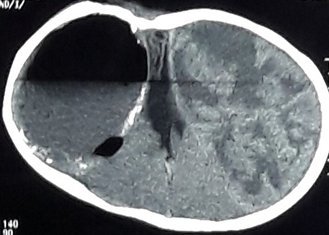
Giant subdural empyema

